# A New Technique for Aortic Annular and Outflow Enlargement: Combined Y-Incision and Nicks Procedures

**DOI:** 10.1016/j.atssr.2024.04.011

**Published:** 2024-04-27

**Authors:** Kosuke Nakamae, Hiroshi Niinami, Satoru Domoto, Takeshi Shinkawa, Kozo Morita

**Affiliations:** 1 Department of Cardiovascular Surgery, Tokyo Women’s Medical University, Tokyo, Japan

## Abstract

In the valve-in-valve transcatheter aortic valve replacement (ViV-TAVR) era, implanting a larger-sized valve during the initial aortic valve replacement is important. For smaller aortic annuli, combining aortic annular and left ventricular outflow tract (LVOT) enlargement is essential. The Y-incision procedure helps achieve implantation of a 2-size larger valve. However, it can lead to size discrepancies between the valve and the LVOT, thus resulting in a residual pressure gradient, and the risk of coronary obstruction after ViV-TAVR remains because the initial surgical valve is implanted tilted inward. To resolve these concerns, we combined the Y-incision and Nicks procedures.

Valve-in-valve transcatheter aortic valve replacement (ViV-TAVR) is becoming increasingly common, and younger patients have been choosing bioprosthetic valves over mechanical valves to avoid using anticoagulant agents during the initial surgical aortic valve replacement (SAVR). Therefore, a implantation of a larger-sized valve is inevitable and important. With smaller aortic annuli, combined aortic annular enlargement is necessary to ensure an adequate effective orifice area.

Bo Yang^1^ reported that the Y-incision procedure facilitates the safe implantation of 2-size larger valves and prevents prosthesis-patient mismatches (PPMs). However, in patients with small left ventricular outflow tracts (LVOTs), a size discrepancy between the valve and the LVOT can arise and result in a residual pressure gradient (PG). Furthermore, the risk of coronary obstruction after ViV-TAVR increases when the initial surgical valve is too large and tilted inward. Thus, we combined the Y-incision and Nicks procedures to address these challenges.

## Technique

**Figure 1:**
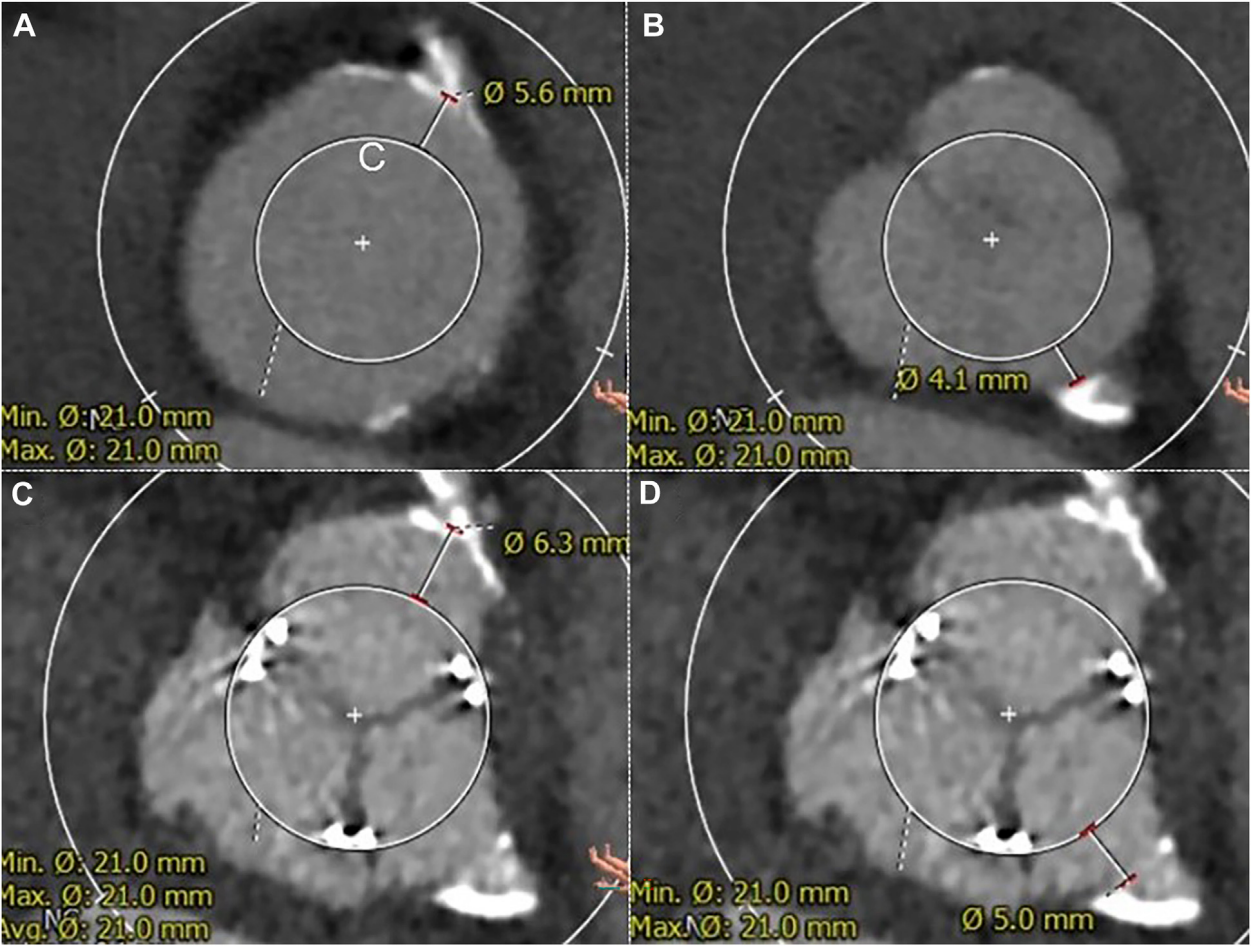
Preoperative computed tomography showing that the virtual distances from the surgical valve to the (A) right and (B) left coronary arteries are 5.6 mm and 4.1 mm, respectively. Postoperative computed tomography showing that the virtual distances from the surgical valve to the (D) right and (E) left coronary arteries are 6.3 mm and 5.0 mm, respectively. (Avg., average; Max., maximum; Min., minimum.)

A 59-year-old woman with aortic regurgitation and an ascending aortic aneurysm required surgery (Video 1). The patient had a small stature (body surface area, 1.07 cm^2^), a small aortic annulus (preoperative annulus area, 258.7 mm^2^; area-derived diameter, 18.1 mm), and a small LVOT (area, 240.3 mm^2^) (Supplemental Figures 1A, 1B). Furthermore, she had an intellectual disability that hindered warfarin intake, and using a mechanical valve was inadvisable. Therefore, SAVR using a bioprosthetic valve with aortic annular enlargement was planned. We estimated that if a 21-mm valve was implanted, the virtual distance from the surgical valve to the coronary artery would be sufficiently secured (Figures 1A, 1B).

**Figure 2:**
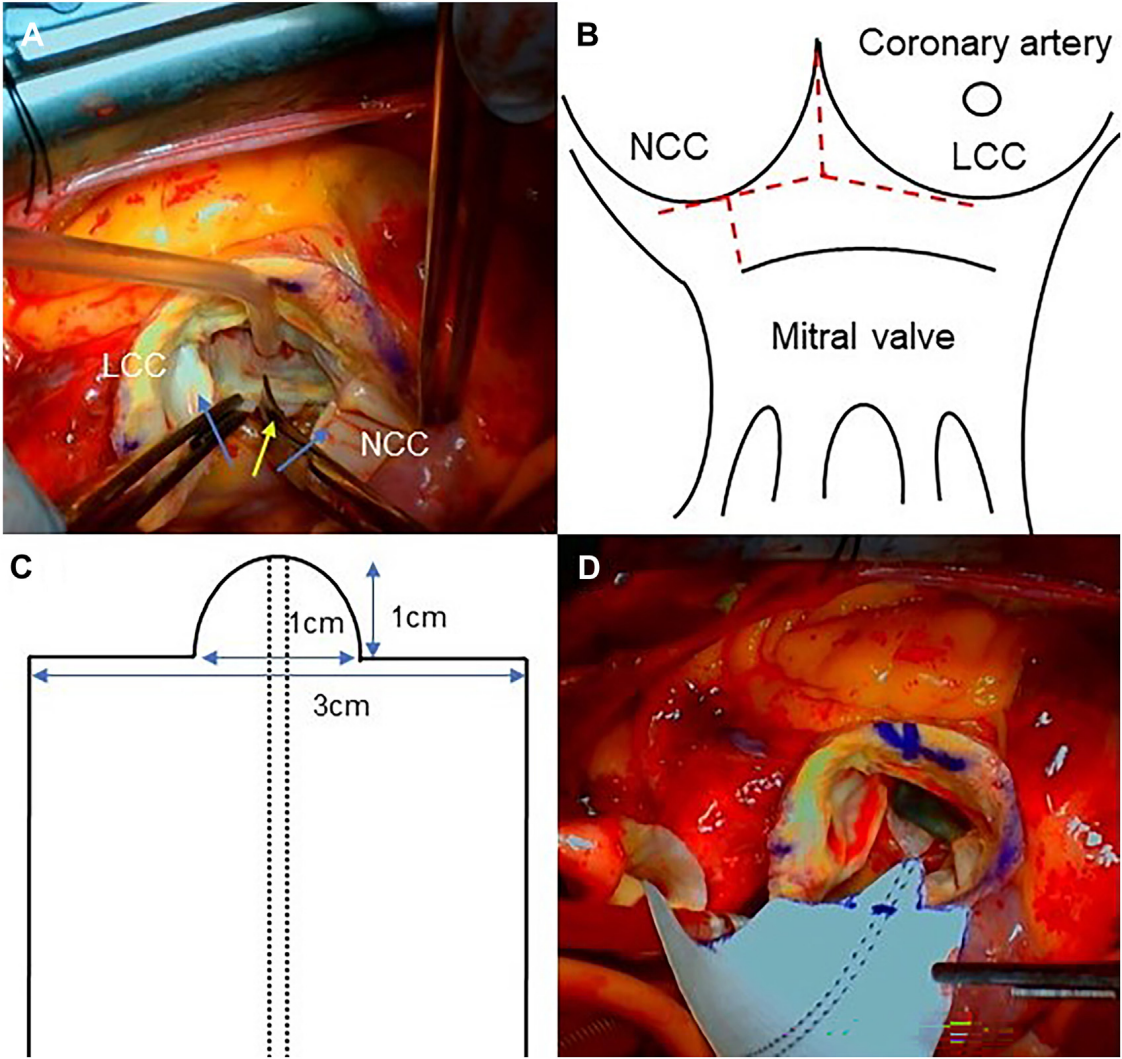
(A and B) The intraoperative findings and schematic illustration show the combination of the Y-incision and the Nicks procedures. An incision was made between the left coronary cusp (LCC) and the noncoronary cusp (NCC) up to the commissure, and a 20-mm incision was made just below the annulus on both sides of the coronary cusps (blue arrows). In addition, a 7-mm incision was made from the NCC sinus across the mitral annulus (yellow arrow). (C and D) The incision measured 3 cm in length and 1 cm in height, and a Hemashield Dacron patch (Boston Scientific) was trimmed to match the shape shown.

**Figure 3:**
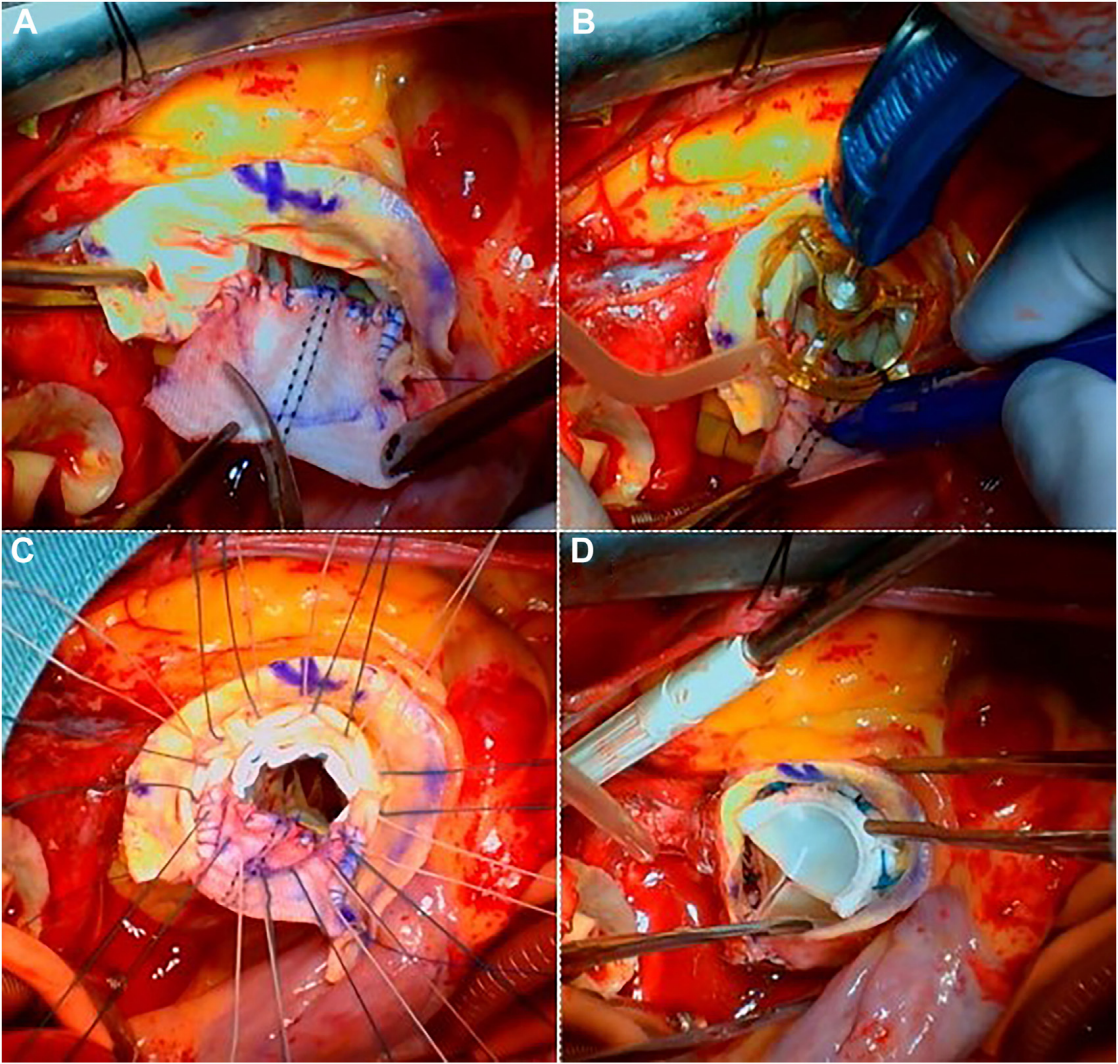
(A) The distal end of the Hemashield Dacron patch (Boston Scientific) was resected in a triangular shape. (B) The upsized valve sizer was placed in the enlarged aortic annulus, and the cuff line was marked to provide guidance for placing the valve suture. (C) The pledgeted sutures were placed along the cuff marker, thus securing the valve to the annulus and the Dacron patch. (D) The distal end of the Dacron patch was sewn to the aortic wall.

Similar to the Y-incision procedure, an incision was made between the left coronary cusp and the noncoronary cusp up to the commissure after the aortic valve was resected. Subsequently, a 20-mm incision was made just below the annulus on the coronary cusps bilaterally. Additionally, a 7-mm incision was made across the noncoronary sinus to the mitral annulus (Figures 2A, 2B). A Hemashield Dacron patch (Boston Scientific) was trimmed to a 3-cm length, with the tip protruding approximately 1 cm from the center of the patch (Figure 2C). The patch was sewn using 4-0 polypropylene (Prolene, Ethicon) running sutures between the left and right fibrous trigones along the mitral annulus and the trimmed patch (Figure 2D). The distal end of the Dacron patch was resected in a triangular shape (Figure 3A). The cuff line was marked with an upsized valve sizer to guide the valve suture (Figure 3B), and pledgeted valve sutures using noneverting mattress sutures were placed along the cuff marker (Figure 3C). A 21-mm Inspiris Resilia valve (Edwards Lifesciences) was implanted, and the distal end of the Dacron patch was sewn to the aortic wall (Figure 3D).

Postoperative echocardiography showed good hemodynamics, with a peak velocity of 1.6 m/s, a mean PG of 6.3 mm Hg, and an effective orifice area of 1.1 cm^2^. The postoperative annulus and LVOT area were sufficiently enlarged to 263.3 mm^2^ (Supplemental Figures 1C, 1D), and the distance from the valve to the coronary artery was secured sufficiently (Figures 1C, 1D).

## Comment

With the increase in ViV-TAVR procedures, the demand for SAVRs with aortic annular enlargement is also rising, to prevent not only PPM but also other complications after ViV-TAVRs. Worse outcomes have been reported in patients with small initial surgical valves, with PPM and high postprocedural gradients after ViV-TAVRs.^2^ The Y-incision procedure, which involves making a Y-incision at the aortomitral curtain and placing a rectangular patch, enables us to implant 2-size larger valves without violating the mitral valve.^1^ Despite the simplicity of the Y-incision procedure to easily enlarge the aortic annulus and implant a larger-sized valve, it enlarges only the supraaortic annular region and not the subaortic annular region or the LVOT. In patients with a small LVOT, a potential size discrepancy with the implanted valve and the LVOT may occur, resulting in residual PGs and tilted valve implantation, leading to coronary artery obstruction after ViV-TAVRs. Therefore, the basal ring needs to be incised to enlarge the aortic annulus and LVOT during the initial SAVR with aortic annular enlargement. However, the Nicks procedure can enlarge the aortic annulus by only 1 size up at most,^3^ and the Manouguian procedure requires incision of the left atrium and mitral valve that causes mitral insufficiency.^4^ A combination of the Y-incision and Nicks procedures could maximize the benefits.

An appropriate-sized valve should be implanted to avoid coronary artery obstruction after ViV-TAVRs. In this case, we preoperatively estimated that if a 21-mm, 2-size larger valve could be implanted, PPM could be adequately avoided, and it could reduce the risk of coronary obstruction after ViV-TAVRs. As assumed, postoperative computed tomography showed that the valve was coaxially implanted (Supplemental Figure 2), and the distance from the valve to the coronary artery increased.

This report describes combined Y-incision and Nicks procedures, which enlarged the aortic annulus and LVOT readily and safely, thereby ensuring better hemodynamics and safer coronary artery protection after ViV-TAVRs.
