# Supplementary material for: A New Technique for Aortic Annular and Outflow Enlargement: Combined Y-Incision and Nicks Procedures
Source: Ann Thorac Surg Short Rep. 2024 Apr 27;2(4):799–803. doi: 10.1016/j.atssr.2024.04.011 (PMC11708572; doi:10.1016/j.atssr.2024.04.011)

Supplemental Figure 1. (A) Preoperative computed tomography showing the area derived annulus diameter of 18.1 mm (the short axis of 14.9 mm), and (B) the area derived LVOT diameter of 17.5 mm (the long axis of 22.8 mm^2^). (C) Postoperative computed tomography showing the area derived annulus diameter of 19.1 mm (the short axis of 18.8 mm), and (D) the area derived LVOT diameter of 18.3 mm (the long axis of 23.0 mm^2^).

LVOT, left ventricular outflow tract


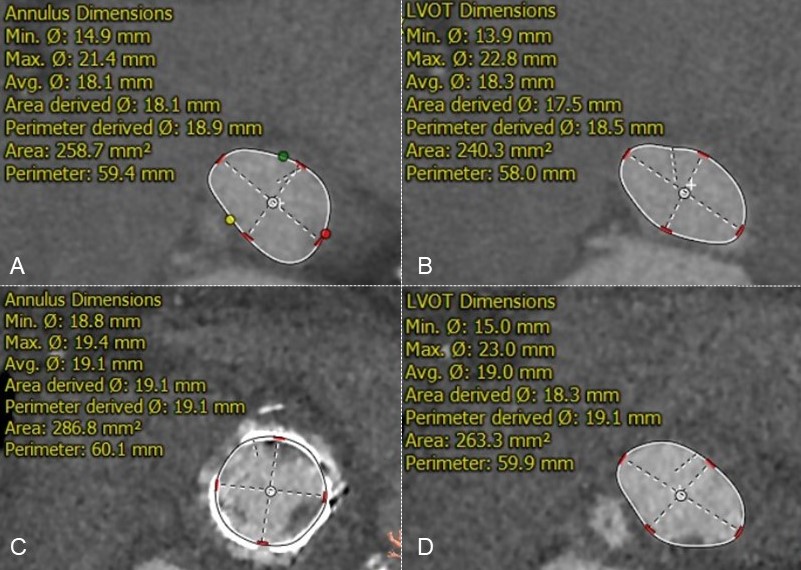


Supplemental Figure 2. Postoperative computed tomography showing the coaxially implanted valve (A), the angle of the valve relative to the aortic annulus of 2°(B), and the virtual valve-in-valve with adequate distance from the coronary arteries (C).


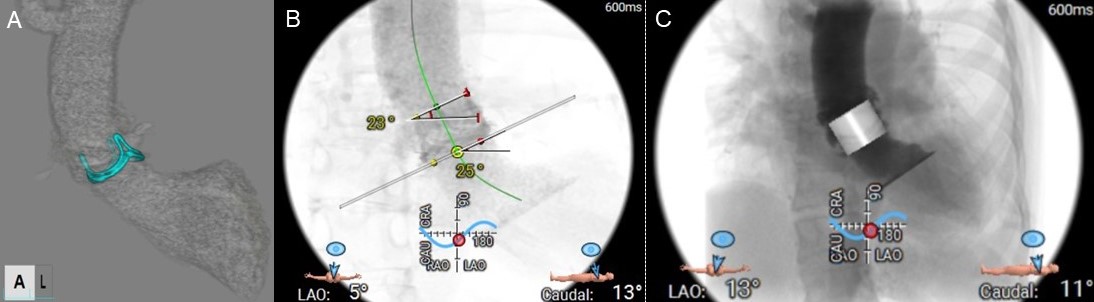

Supplement: Supplemental Figures [file mmc2.docx]
